# Data analytics and artificial intelligence in predicting length of stay, readmission, and mortality: a population-based study of surgical management of colorectal cancer

**DOI:** 10.1007/s12672-022-00472-7

**Published:** 2022-02-28

**Authors:** Shamsul Masum, Adrian Hopgood, Samuel Stefan, Karen Flashman, Jim Khan

**Affiliations:** 1 Faculty of Technology, University of Portsmouth, Portland Building, Portland Street, Portsmouth, PO1 3AH UK; 2 Colorectal Department, Portsmouth Hospitals University NHS Trust, Southwick Hill Road, Portsmouth, PO6 3LY UK; 3 Faculty of Science & Health, University of Portsmouth, St Michael’s Building, White Swan Road, Portsmouth, PO1 2DT UK

**Keywords:** Colorectal cancer, Colorectal surgery, Prediction, Predictor variables, Length of stay, Readmission, Mortality, Data analytics, Artificial intelligence, Machine learning

## Abstract

Data analytics and artificial intelligence (AI) have been used to predict patient outcomes after colorectal cancer surgery. A prospectively maintained colorectal cancer database was used, covering 4336 patients who underwent colorectal cancer surgery between 2003 and 2019. The 47 patient parameters included demographics, peri- and post-operative outcomes, surgical approaches, complications, and mortality. Data analytics were used to compare the importance of each variable and AI prediction models were built for length of stay (LOS), readmission, and mortality. Accuracies of at least 80% have been achieved. The significant predictors of LOS were age, ASA grade, operative time, presence or absence of a stoma, robotic or laparoscopic approach to surgery, and complications. The model with support vector regression (SVR) algorithms predicted the LOS with an accuracy of 83% and mean absolute error (MAE) of 9.69 days. The significant predictors of readmission were age, laparoscopic procedure, stoma performed, preoperative nodal (N) stage, operation time, operation mode, previous surgery type, LOS, and the specific procedure. A BI-LSTM model predicted readmission with 87.5% accuracy, 84% sensitivity, and 90% specificity. The significant predictors of mortality were age, ASA grade, BMI, the formation of a stoma, preoperative TNM staging, neoadjuvant chemotherapy, curative resection, and LOS. Classification predictive modelling predicted three different colorectal cancer mortality measures (overall mortality, and 31- and 91-days mortality) with 80–96% accuracy, 84–93% sensitivity, and 75–100% specificity. A model using all variables performed only slightly better than one that used just the most significant ones.

## Introduction

Colorectal cancer is the third most common cancer by incidence, with over 1.8 million new cases in 2018, and the second most common cause of cancer death when the sexes are combined [1]. Around 147,950 and 42,300 new cases of colorectal cancer were predicted for the USA and UK respectively in 2020 [2, 3]. Moreover, it is estimated that there will be around 2.4 million new cases worldwide in 2035 [4]. The reasons for this growth in cases are unclear and are the subject of clinical and basic research [5].

This article considers a range of factors affecting the patient outcomes after surgery, covering both the patient’s individual characteristics and the nature of the surgery. The patient characteristics include performance status (ASA grade) and BMI, reflecting prior work that shows the effects of obesity on a range of conditions, including cancers [6].

LOS, readmission and mortality are essential proxies of quality of care in surgery [7–10]. Shorter LOS could potentially minimise healthcare costs, free up hospital beds, improve productivity, reduce the risk of nosocomial infections and improve quality of life. Increased readmission rates have a huge impact on healthcare costs. Readmission within 30 days annually costs around 40 billion [11, 12]. Moreover, a higher readmission rate indicates poor discharge planning and post-operative morbidity, with a clinical and psychological impact on the patient. Overall mortality rates following colorectal surgery range from 1 to 16.4% [13–15]. The National Cancer Intelligence Network found that the 30-day post-operative mortality rate is falling across England, with the overall post-operative mortality rate of 6.7%. This rate improved over the study period from 6.9% in 1998 to 5.9% in 2006 [16]. The National Bowel Cancer Audit in their recent annual report found a downward trend in 90-day post-operative mortality with a rate of 3.0%. The study also showed that 90-day post-operative mortality has reduced from 2.3% in 2013/14 to 1.7% in 2017/18 for elective surgery and from 14.2 to 11.5% for emergency surgery [17].

The economic impact of colorectal cancer on healthcare systems is intense. The US is expected to spend around US$17.41 billion on colorectal cancer, and approximately US$4.2 billion in productivity lost to deaths related to colorectal cancer in 2020 [18]. In the UK, the cost of diagnosing and treating colorectal cancer patients is significant (€40,000 per case) [19] and exacerbates funding constraints on the National Health Service (NHS) [8]. With limited resources and a finite surgical bed capacity in many hospitals, it is extremely important to know the expected LoS, readmission rate and mortality after elective CRC surgery.

In recent times, Artificial Intelligence (AI) and Machine Learning (ML) techniques have shown great promise in the diagnosis and prognosis of various diseases and health conditions [20, 21]. ML aims to discover patterns from data without explicit programming. ML algorithms are used to model and learn important properties from data, including the stochastic dependency between a set of input and output variables. ML is a data-driven technique that has the benefit of integrating multiple risk factors into a prediction model [22]. Meanwhile, ML techniques have been found useful in detecting colorectal cancer in advance where the model was constructed with blood cell count, age and sex as input features [23].

An accurate prediction of LOS, readmission and mortality would help healthcare professionals with planning, decision making and building strategies. This will eventually lead to improved patient care and prevent readmission and mortality after discharge [24]. A prediction model that could predict readmission accurately would help healthcare professionals to intervene in readmission scenarios and provide better patient care. A model with the ability to predict the mortality would be valuable to patients, surgeons and healthcare institutions. An accurate mortality prediction model would contribute to patient risk stratification, preoperative consultation with the patient and family members, decision-making process, consent and professional accountability.

This study has investigated the scope of AI and data analytics in predicting LOS, readmission and mortality in colorectal cancer patient’s treated in a large NHS trust. Predictor variables of LOS, readmission and mortality have been explored using data analysis to determine which predictors are most important. Machine learning algorithms were then investigated as predictive tools. A comparison has been made between using all variables as predictors for machine learning versus using just the most significant variables.

## Methodology

Codes and findings of feature selection experiments are available in the github repository [25]. The uploaded file in the repository is currently protected with a password, ‘colorectalai’.

### Data

Records of a prospectively maintained colorectal cancer database by the Colorectal Department in a large NHS Trust were examined. The dataset contains 4336 patients who underwent colorectal cancer surgery between 2003 and 2019. The 47 patient parameters/variables included demographics, peri- and post-operative outcomes, surgical approaches, complications and mortality (see Table 1). Table 2 shows various classes associated with variables in the dataset. The table shows the four different classes of procedure, 17 different classes of specific procedure, two classes of laparoscopic type, surgical approach (open or laparoscopic), robotic (yes/no), operation mode (elective or emergency), and complications (yes/no). Both intra- and post-operative complications are considered together.

**Table 1:** Available variables in the dataset

Sex	Complication (y/n)	pN stage
ASA	Additional procedures	Resection margin
Age	Stoma formation	Radio therapy
BMI	Robotic (y/n)	Chemo therapy
Cancer site	Surgical approach	misc_info
TumICD10	Laparoscopic type	Mortality
Preoperative T stage	Curative surgery	Death_date
Preoperative nodal stage	Operation time	Death_check
Preoperative M stage	Blood loss	Private_pt
Previous abdominal surgery	LOS	Local recurrence date
Previous surgery type	Readmit < 31 days	Distant recurrence date
Operation mode	REOP < 31 days	Distant recurrence days
Resection (y/n)	Complication misc	Local recurrence days
Procedure type (4 classes)	LN harvest	Mortality < 31 days
OPCS4	LN positive	Mortality < 91 days
Specific procedure (17 classes)	pT stage

**Table 2:** Classes associated with variables in the dataset

Variables	Class	
Procedure types	(1) Closed without procedure	
	(2) Stoma only	
	(3) Bypass/stent	
	(4) Excision	
Specific procedure	(1) Right hemicolectomy	(10) TEMS
	(2) Extended right hemicolectomy	(12) Polypectomy
	(3) Transverse colectomy	(11) Stent
	(4) Left hemicolectomy	(13) Laparotomy only
	(5) Sigmoid colectomy	(14) Laparoscopy only
	(6) Anterior resection	(15) Stoma only
	(7) APER	(16) Other
	(8) Hartmann’s procedure	(17) Bypass
	(9) Trans anal resection of tumour	
Laparoscopic type	(1) Laparoscopic completed	
	(2) Laparoscopy converted to open	
Surgical approach	(1) Open operation	
	(2) Laparoscopic operation	
Robotic	(1) Yes	
	(2) No	
Operation mode	(1) Elective	
	(2) Emergency	
Complication	(1) Yes	
	(2) No	

Descriptive statistics of some variables that summarise the central tendency, dispersion and shape of the distribution of a dataset, excluding NaN values, can be seen in Table 3. There were 2494 male and 1942 female patients in the dataset. Among these 4336 cases, 74% (3209) were curative, 13.35% (579) were palliative, and 6.53% (283) were uncertain. 80% (3475) of the surgeries performed were elective in comparison to 18% (782) of emergency. Assistance from the robot was considered for 8.9% (388) of the cases. The laparoscopic approach was applied to 57.45% (2491) of the cases, whereas open surgery was used in 35.79% (1552). The 30-day readmission rate was 7.4%. Moreover, the 30- and 90-day mortality was 3.39% (147) and 5.93% (257) respectively.

**Table 3:** Statistics of selected variables from the dataset

Descriptive statistics	Age	Operation time	Blood loss	BMI	LOS	Mortality days
Mean/average	70.24	181.18	66.16	26.85	11.26	1131.32
Standard deviation	11.61	93.15	78.89	4.26	12.12	1152.21
Minimum value	24.00	0.00	0.00	13.50	0.00	0.00
25th percentile	63.00	160.00	50.00	25.00	5.00	245.25
50th percentile	72.00	181.18	66.16	26.85	8.00	724.50
75th percentile	79.00	215.00	66.16	28.00	13.00	1654.00
Maximum value	97.00	690.00	1200.00	78.50	252.00	5542.00

#### Data processing

The problems of missing values and mixed data types were dealt with using appropriate techniques and with the help of medical domain knowledge through discussions with clinicians. Following clinical discussion, missing values were filled with different techniques (see Table 4). The dataset consists of some columns where data types are mixed. For example, Sex and TumICD10 variables have both text and numeric data. In order to fit them to machine learning algorithms, all these mixed data types are converted to numeric data using the Pandas Series.str.replace() method [26]. Moreover, there are some columns that comprise text values only (e.g., Robotic, Radiotherapy). All these columns that consist of text data are passed through the LabelEncoder methods of scikit learn [27] to convert them to numeric data.

**Table 4:** Alternative techniques to fill missing values with medical domain knowledge

Variables	No of missing values	Methods to fill missing values
Sex	0	–
ASA	593	Mode
LOS	179	Mean
Operation mode	79	Min
Curative surgery	265	Min

### Prediction model building

#### Model for LOS

**Fig. 1:**
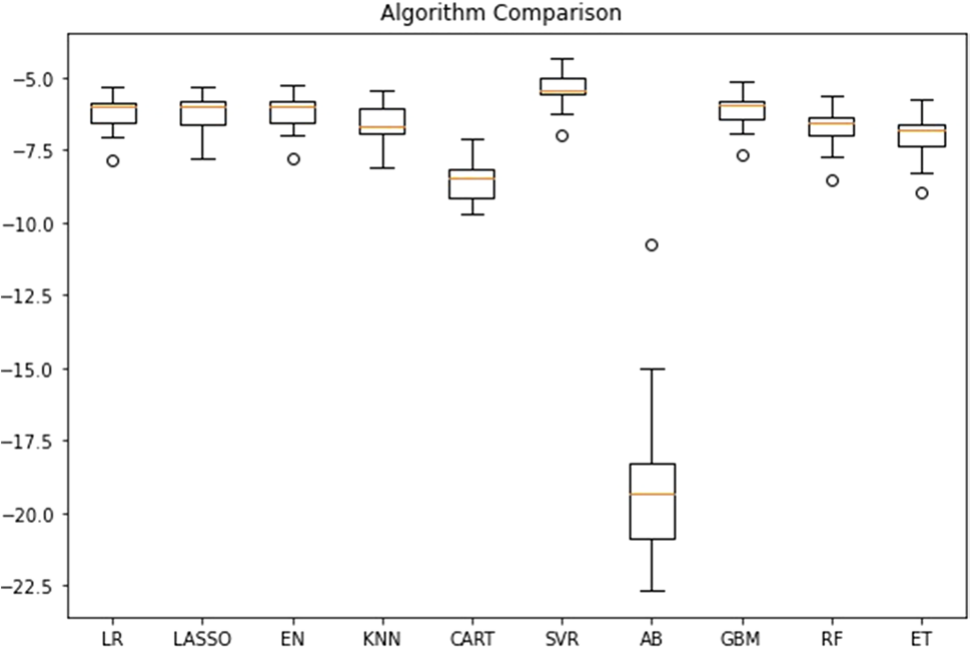
Comparison of algorithms for modelling the data

Regression predictive modelling was performed to predict the LOS. The data were power-transformed to make them more Gaussian-like [28]. Then the data were discretized to map numerical variables onto discrete values. Such mapping creates a high-order ranking of values that can smooth out the relationships between observations and is found useful for machine learning [29]. A 10-fold cross-validation technique [30, 31] was used for splitting the training and test data. Different algorithms were compared to find the optimal model for LOS prediction. A negative mean absolute error was used as the evaluation metric to compare different algorithms. Comparison between algorithms shows that support vector regression (SVR) outperformed the other algorithms (see Fig.  1). Following this finding, different parameters of the SVR algorithms (see Table 5) were tuned using the GridSearchCV technique [32]. The model was trained with the training dataset and tested on the test dataset. Finally, different evaluation metrics, namely root-mean-square error (RMSE), mean absolute error (MAE), and accuracy, were used to evaluate the model. Data analysis of different variables in predicting LOS was also conducted.

**Table 5:** Tuning the parameters of the SVR algorithm

Parameters	Range	Best parameters
kernel	[‘linear’, ‘poly’, ‘rbf’]	rbf
C	[1, 5, 10, 15]	10
degree	[1, 2, 3]	1
gamma	[‘scale’, ‘auto’]	scale
coef0	[0, .01, 0.1]	0.01
epsilon	[0.1, 0.5, 0.9]	0.9

#### Model for readmission

Classification predictive modelling was performed to predict the readmission. Models with Random Forest (RF), K Nearest Neighbor (KNN), Support Vector Machine (SVM), Multilayer Perceptron (MLP), and Bidirectional Long Short-Term Memory (BI-LSTM) algorithms were compared for readmission prediction. For the BI-LSTM algorithm, the data are reshaped following the work of Masum et al. [33, 34] as the LSTM based RNN requires input to be in a matrix with the dimensions: [samples, time steps, features]. The model with BI-LSTM algorithm has been designed so that the network structure consists of three hidden layers with 100 LSTM units, then an output layer with the sigmoid activation. The network also represented binary crossentropy as a loss function, ADAM algorithm [35] as an optimizer, and accuracy as metrics. The network has been fitted with 20 epochs and a batch size of 2. 80% of data of the dataset was used for training and 20% was used for testing purpose.

#### Model for mortality

Classification predictive modelling was performed to predict the mortality. Models with Random Forest (RF), K Nearest Neighbor (KNN), Support Vector Machine (SVM), Multilayer Perceptron (MLP), and Bidirectional Long Short-Term Memory (BI-LSTM) algorithms were compared for mortality, and 31- and 91-days mortality prediction. The model structure for readmission prediction mentioned in Sect. 2.2.2 was used for mortality prediction scenarios.

### Comparing variables

The variable that needs to be predicted is known as the target variable and the variables that are used to predict the target variable known as features. Identifying the best features is an important task [36]. A large number of features could lead to complex model, long training time, the curse of dimensionality, noise addition, overfitting etc. On the other hand, a smaller number of variables could lead to the exclusion of relevant variables. ExtraTreeRegressor [37], ExtraTreesClassifier [37], LassoCV [38] and Correlation Matrix analysis with Heat Map of scikit-learn [27] have been considered for feature selection. Moreover, in all prediction cases, 80% of the dataset was used for training and 20% was used for testing purposes.

#### Feature selection for LOS

Extra Tree Regressor showed that Age, BMI, Surgical approach, Operation time, ASA, Blood loss, Preoperative T stage, Stoma formation, Sex and Preoperative nodal stage were the most crucial features in predicting LOS (see github repository [25]). In contrast, a LASSO algorithm showed that Surgical approach, Sex, Chemotherapy, ASA, Operation mode, Stoma formation, TumID10, Procedure type, Additional procedures, Radiotherapy, Preoperative T stage, Age and, Cancer site were the most important features (see github repository [25]). Moreover, the features explored through a correlation matrix with heat map has found Surgical approach, ASA, Age, Operation mode, Complication, Stoma formation, Chemotherapy, TumID10, Preoperative T stage as essential features (see github repository [25]). Following the findings from these techniques, we considered Age, ASA, Surgical approach, Stoma formation, Preoperative T stage, Chemotherapy, Operation mode, TumID10, Cancer site, and Radiotherapy as the selected features for predicting LOS.

#### Feature selection for readmission

Extra Tree Classifier showed that Surgical approach, Operation time, LOS, BMI, Age, ASA, Blood loss, Preoperative T stage, Stoma formation were the most crucial features in predicting readmission (see github repository [25]). In contrast, a LASSO algorithm showed that Surgical approach, Operation mode, Previous surgery type, Stoma formation, Preoperative nodal stage, the Specific procedure, ASA, BMI, Age, Sex, LOS and Cancer site were the most important features (see github repository [25]). Moreover, features explored through a correlation matrix with heat map have found Surgical approach, Age, Preoperative nodal stage, Pre abdominal surgery, Previous surgery type, Operation mode, Complication, Additional procedures, Robotic, Curative Surgery, Operation time and LOS as essential features (see github repository [25]). Following the findings from these techniques, we considered Age, Surgical approach, Stoma formation, Preoperative nodal stage, Operation time, Operation mode, Previous surgery type, LOS, and the Specific procedure as the selected features for predicting readmission.

#### Feature selection for mortality

Extra Tree Classifier showed that Age, LOS, Curative Surgery, BMI, ASA, Operation time, Surgical approach, Chemotherapy, Radiotherapy, Operation mode were the most critical features in predicting mortality (see github repository [25]). A LASSO algorithm showed that Curative Surgery, Chemotherapy, Preoperative T stage, Operation mode, ASA, Preoperative M stage, Stoma formation, Age, the Specific procedure, LOS, OPCS4, BMI, Cancer site, Previous surgery type, and Surgical approach were the most important features (see github repository [25]). Moreover, features explored through a correlation matrix with heat map has found ASA, Age, BMI, Preoperative T stage, Preoperative M stage, Operation mode, Procedure type, Complication, Stoma formation, Radiotherapy, Chemotherapy, Surgical approach, Curative Surgery and LOS as essential features (see github repository [25]). Following the findings from these techniques, we considered Age, ASA, BMI, Chemotherapy, Preoperative M stage, Surgical approach, LOS, Curative Surgery, Preoperative T stage and Stoma formation as the selected features for predicting mortality. For 31 days mortality prediction, we selected Chemotherapy, Additional procedures, Operation mode, Complication, Previous abdominal surgery, Previous surgery type, Surgical approach, Curative Surgery, Age and Resection as selected features. In contrast, for 91 days mortality prediction we selected LOS, Additional procedures, Curative Surgery, Complication, Previous surgery type, Pre abdominal surgery, Procedure type, Operation mode, Surgical approach and Resection as the selected features.

#### Model for comparing variables in LOS, readmission and mortality prediction

The model with the SVR algorithm mentioned in Sect. 2.2.1 was used in comparing variables in LOS prediction. To compare variables in readmission prediction, the model with BI-LSTM algorithm mentioned in Sect. 2.2.2 was used. Moreover, the model with BI-LSTM algorithm used in Sect. 2.2.3 was used in comparing variables in all scenarios of mortality prediction.

## Results

### LOS prediction

**Fig. 2:**
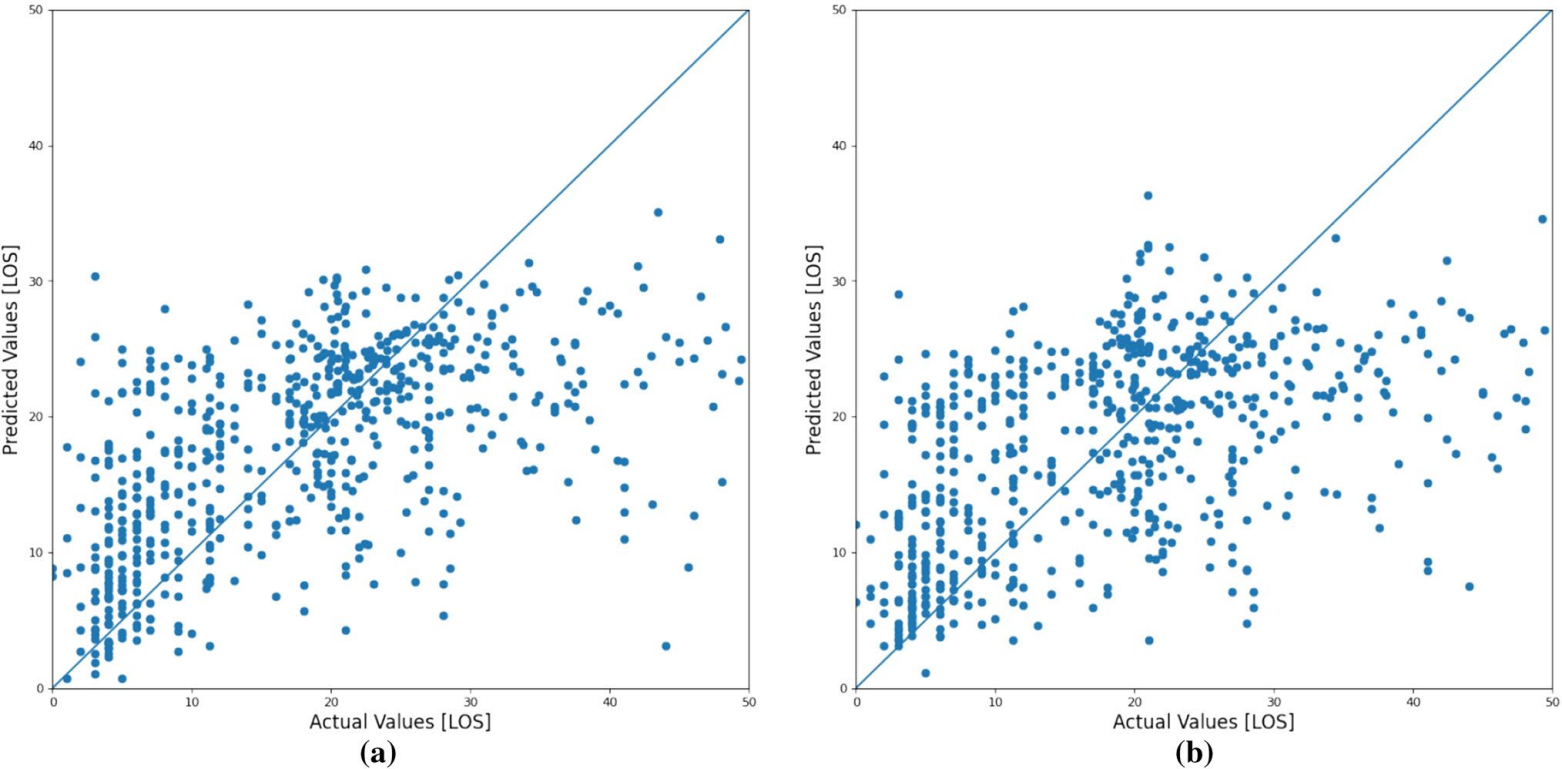
LOS prediction: **a** represents the actual versus predicted LOS when considering all variables and **b** represents the actual versus predicted LOS when considering only selected variables

The model with a SVR algorithm predicted the LOS with an MAE and RMSE of 9.69 and 12.52 respectively. Figure  2 shows how the model predicted the LOS. The regression outcome of the model is then converted to binary class using these conditions:

- True class (1) = if patient’s stay is correctly predicted ≤ 6 days
- True class (1) = if patient’s stay is correctly predicted > 6 days
- False class (0) = if patient’s stay is incorrectly predicted ≤ 6 days
- False class (0) = if patient’s stay is incorrectly predicted > 6 days

Converting the regression outcome to a binary class helps to calculate the accuracy of the model and accuracy of 83.21% was recorded when all available variables were considered as inputs.

### Data analysis of LOS

**Fig. 3:**
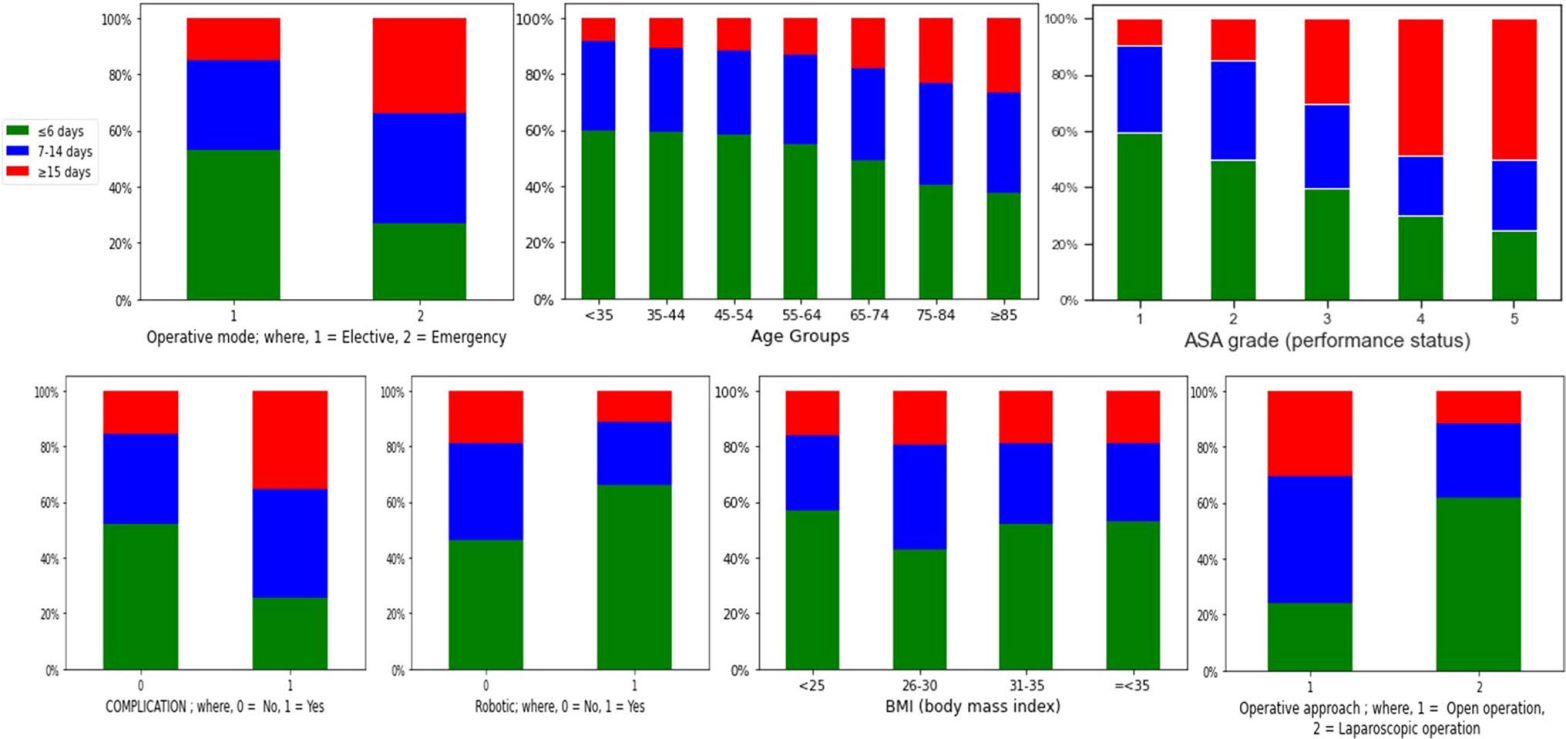
Data analysis of LOS

Data analysis of different variables for LOS shows that age groups, ASA grade, whether a robotic surgery was performed or not, whether a laparoscopic operation was performed or not, operation mode and complications all have a significant impact in LOS prediction. LOS was grouped into three categories (≤ 6, 7–14 and ≥ 15 days). Different variables were also categorised accordingly (see Fig.  3). Data analysis of different variables in relation to LOS indicates that:

- The number of patients who had a LOS of 15 days and over increased with age. Moreover, the number of patients who had a LOS of 6 days and less decreased with age.
- Patients with better performance status (ASA grade) have shorter hospital stays compared with patients with poor performance status (ASA grade).
- Observation of the surgical approach shows that a laparoscopic operation leads to shorter LOS compared with open operation.
- Patients with an emergency operation were more likely to have an increased LOS compared with an elective operation.
- Patients with a complication were more likely to have an increased LOS compared with patients without difficulty.
- Patients’ stays in hospital are shorter when robotic surgery was performed in comparison with non-robotic surgery.
- It was also observed that BMI is not a good indicator of LOS.

### Readmission prediction

**Fig. 4:**
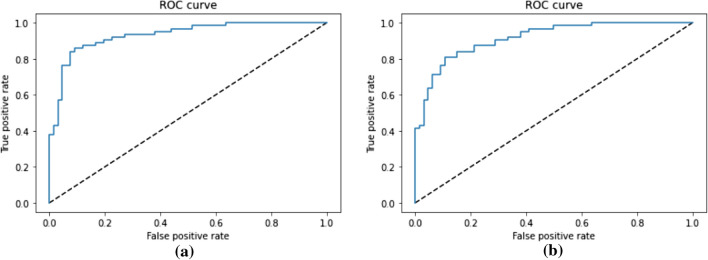
Readmission prediction: **a** represents the ROC CURVE when considering all variables and **b** represents the ROC CURVE when considering only selected variables

The dataset contains 321 readmission cases. Different algorithms were compared in readmission prediction, and it was found that the model with a BI-LSTM algorithm outperformed the other algorithms (see Table 6). The model with a BI-LSTM algorithm predicted the readmission with an accuracy of 87.5%, a sensitivity of 84.1% and a specificity of 90.9%. ROC curve of the model for predicting readmission is presented in Fig.  4.

**Table 6:** Prediction of readmission with different algorithms

Algorithm	Accuracy	Sensitivity	Specificity
Random Forest	0.768	0.753	0.781
KNN	0.675	0.619	0.728
SVM	0.681	0.482	0.878
MLP	0.740	0.725	0.757
BI-LSTM	0.875	0.841	0.909

### Data analysis of readmission

**Fig. 5:**
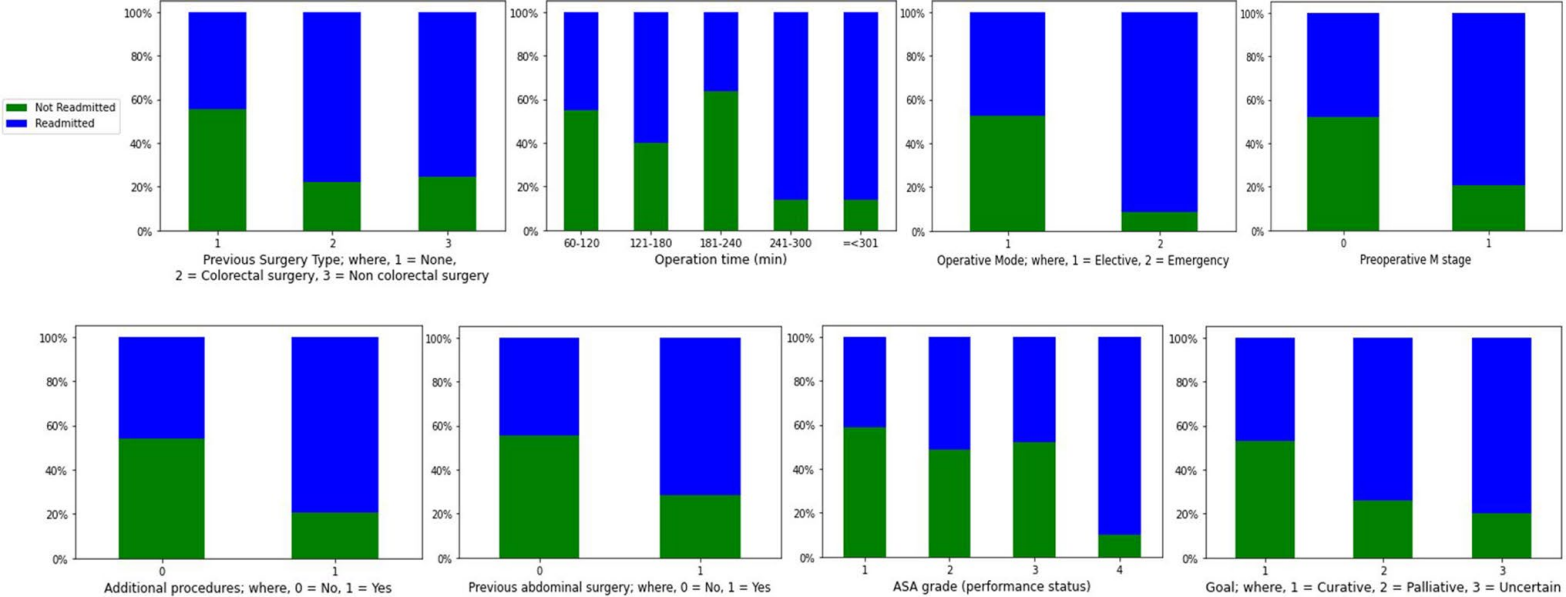
Data analysis of readmission

Data analysis of different variables for readmission shows that the following factors have a strong impact on readmission: ASA grade, operation mode, additional procedure, whether the cancer is curative or not, previous abdominal surgery, types of previous surgery, preoperative M stage and time of the operation. Readmission was grouped into two categories (Readmitted and Not Readmitted). Different variables were also categorised accordingly (see Fig.  5). Data analysis of different variables concerning readmission indicates that:

- A higher percentage of patients are readmitted in a palliative and uncertain scenario compared with a curative scenario.
- Patients with an emergency operation are more likely to be readmitted than those with an elective operation.
- Additional procedures during surgery increase the chance of readmission.
- The readmission rate following colorectal surgery is low for patients with better performance status (ASA grade) compared with poor performance status (ASA grade).
- Previous abdominal surgery before colorectal surgery increases the readmission rate.
- Previous surgery (both bowel and non-chronic groups) prior to colorectal surgery also increases the readmission rate.
- Patients with a preoperative M stage have a high chance of getting readmitted compared with those without.
- A longer operation time increases the patients’ likelihood of readmission.

### Mortality prediction

The model with BI-LSTM algorithms predicted the overall mortality with an accuracy of 80%, a sensitivity of 84.3% and specificity of 75.3% (Table 7). The BI-LSTM algorithms outperformed random forest, KNN, SVM and MLP in predicting mortality prediction. The model with a random forest algorithm predicted the 31 days mortality with an accuracy of 98.9%, a sensitivity of 97.9% and a specificity of 100%. The model with BI-LSTM algorithm also performed well in predicting 31 days mortality with an accuracy of 96.6%, a sensitivity of 93.7% and a specificity of 100%. Moreover, the dataset only contains 146 cases of 31 days of mortality.

**Fig. 6:**
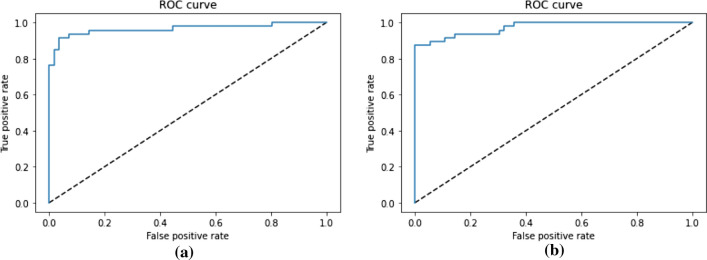
Prediction of 91 days mortality: **a** represents the ROC CURVE when considering all variables and **b** represents the ROC CURVE when considering only selected variables

The model with a BI-LSTM algorithm predicted the 91 days mortality with an accuracy of 94.2%, a sensitivity of 91.4% and a specificity of 96.3% (Table 7). The model with a random forest algorithm performed second-best in predicting 91 days mortality with an accuracy of 87.9%, a sensitivity of 89.4% and a specificity of 86.4%. ROC curve of the model predicting 91 days mortality is presented in Fig.  6. The dataset contains only 257 cases of 91 days of mortality.

**Table 7:** Predictions of mortality, 31 days mortality, and 91 days mortality with different algorithms

Algorithms	Mortality			31 days mortality			91 days mortality		
	Accuracy	Sensitivity	Specificity	Accuracy	Sensitivity	Specificity	Accuracy	Sensitivity	Specificity
Random Forest	0.701	0.590	0.812	0.989	0.979	1	0.879	0.894	0.864
KNN	0.657	0.595	0.719	0.818	0.877	0.760	0.798	0.852	0.744
SVM	0.635	0.679	0.590	0.760	0.918	0.699	0.743	0.898	0.589
MLP	0.740	0.778	0.757	0.900	0.911	0.910	0.852	0.871	0.833
BI-LSTM	0.800	0.843	0.753	0.966	0.937	1	0.942	0.914	0.963

### Data analysis of mortality days

**Fig. 7:**
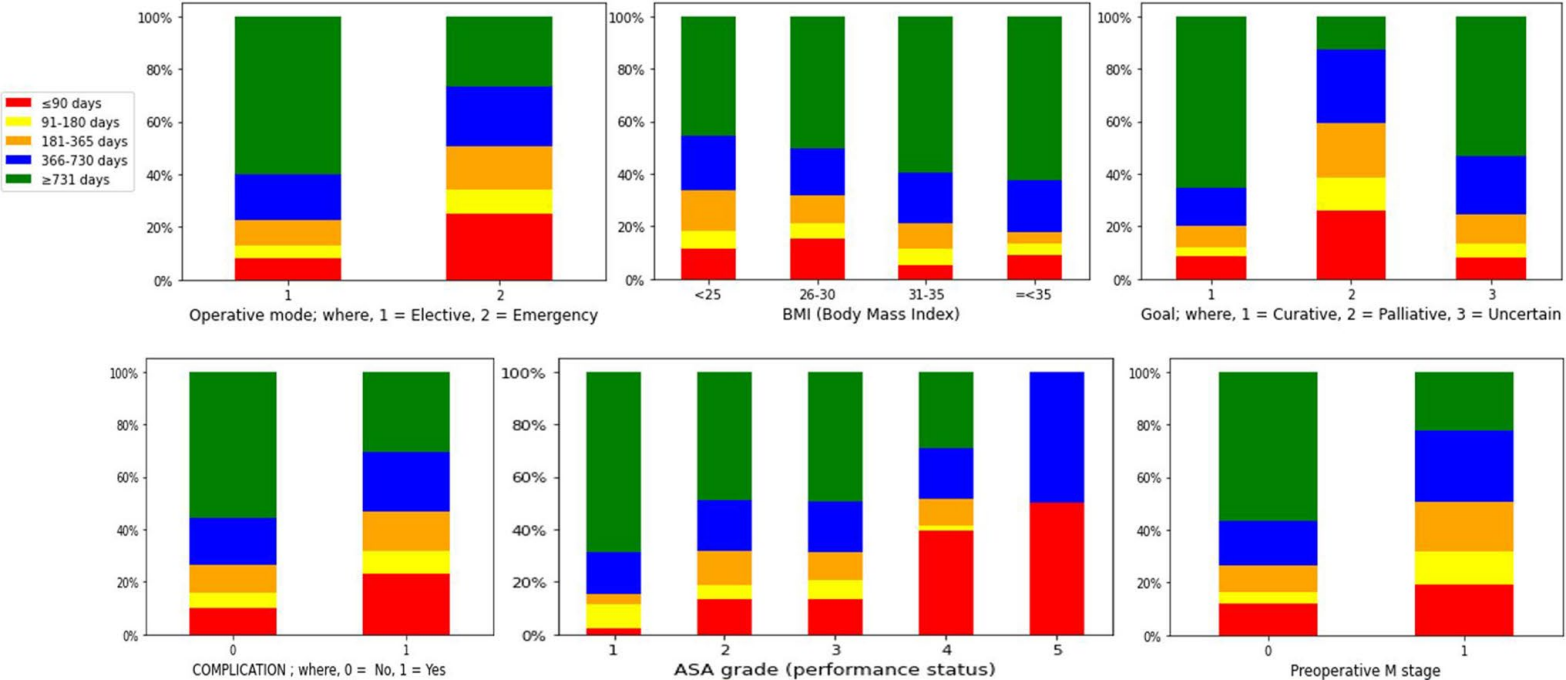
Data analysis of mortality days

Data analysis of mortality days shows that ASA grade, complication, additional procedures, operation mode, whether the cancer is curative or not and preoperative M stage have a strong impact on mortality days. Mortality days were grouped into five categories (≤ 90, 91–180, 181–365, 366–730 and >730 days). Different variables were also categorised accordingly (see Fig.  7). Data analysis of different variables concerning mortality days indicates that:

- Patients with better performance status (ASA grade) live longer than those with poor performance status (ASA grade) following colorectal surgery.
- Complications during colorectal surgery lead to shorter lives compared with no complications. Postoperative complications also have a direct impact on patient survival.
- Patients with a palliative scenario would live for a shorter period compared with a curative and uncertain scenario.
- Patients with preoperative M stage have a reduced life expectancy compared with those without.
- Additional procedures during index operation indicate advanced stage of disease and are associated with prolonged operative time and reduced survival.
- Patients with elective surgery would live longer compared with emergency surgery.
- It was also observed that BMI is not a good indicator of mortality.

### Comparison between all variables and selected variables

The model with the SVR algorithm mentioned in Sect. 2.3.4 was fed with two different types of the dataset to predict the LOS. In one case, all 27 relevant features related to LOS were used as input. In contrast, in the other case, 11 selected variables mentioned in Sect. 2.3.1 were used as the input in predicting LOS. Comparison between the two scenarios can be seen in Table 8. It shows that the model with all variables as features performs only slightly better than the model with selected variables. The model that used all variables as features predicted the LOS with an accuracy of 83.21 %, MAE of 9.69 and RMSE of 12.52. In contrast, an accuracy of 82.61 %, MAE of 10.32 and RMSE of 12.79 were recorded when the model used selected variables. Comparison of actual versus predicted LOS of both cases can be seen in Fig.  2.

**Table 8:** Comparison between the use of all variables versus selected variables in LOS prediction

Evaluation metrics	All variables as feature (27)	Selected variables as feature (10)
RMSE	12.52	12.79
MAE	9.69	10.32
Accuracy	83.21	82.61

Comparison between all variables and selected variables in predicting readmission can be seen in Table 9. It shows that the model mentioned in Sect. 2.3.4 with the use of all 28 variables predicted the readmission scenario with an accuracy of 87.5%, a sensitivity of 84.1% and specificity of 90.9%. This outperforms the model outcome where nine selected variables were considered. The model with selected variables predicted the readmission scenario with an accuracy of 83.7%, a sensitivity of 76.1% and specificity of 90.9%. ROC curve of each scenario is also presented in Fig.  4.

**Table 9:** Comparison between the use of all variables versus selected variables in readmission prediction

Evaluation metrics	All variables as feature (28)	Selected variables as feature (9)
Accuracy	87.5	83.7
Sensitivity	84.1	76.1
Specificity	90.9	90.9

The model mentioned in Sect. 2.3.4 for mortality prediction predicted the mortality with an accuracy of 80%, a sensitivity of 84.3% and specificity of 75.3% when all 29 variables were considered as features. The model performance decreased when the 10 selected variables were considered. An accuracy of 78.4%, sensitivity of 79% and specificity of 77.8% were recorded (see Table 10 ). The model performance does not vary much for 31 and 91 days mortality when these two different sets of data were used for comparison (see Tables 11 and 12). ROC curve of each scenario predicting 91 days mortality is also presented in Fig.  6.

**Table 10:** Comparison between the use of all variables versus selected variables in mortality prediction

Evaluation metrics	All variables as feature (29)	Selected variables as feature (10)
Accuracy	80.0	78.4
Sensitivity	84.3	79.0
Specificity	75.3	77.8

**Table 11:** Comparison between the use of all variables versus selected variables in 31 days mortality prediction

Evaluation metrics	All variables as feature (29)	Selected variables as feature (10)
Accuracy	96.6	96.6
Sensitivity	93.7	93.9
Specificity	100.0	100.0

**Table 12:** Comparison between all variables versus selected variables in 91 days mortality prediction

Evaluation metrics	All variables as feature (29)	Selected variables as feature (10)
Accuracy	94.2	94.1
Sensitivity	91.4	87.2
Specificity	96.3	100.0

## Discussion

### Role of data analytics and machine learning

LOS, readmission and mortality are widely used proxies and quality indicators of care and healthcare spending following colorectal surgery. Accurate prediction of these three proxies remains a crucial challenge after colorectal cancer surgery and would lead to substantial resource implications for clinical and management teams. Consequently, this study has aimed to predict LOS, readmission, and mortality with various machine-learning algorithms. Moreover, different predictor variables were explored and investigated through data analysis and machine learning techniques. A single centre’s data were used for the experiments. The dataset was then processed according to the models’ requirements. Data analysis and feature analysis of the dataset were also performed.

Prior research has used a limited number of variables to investigate LOS, mortality and readmission following CRC surgery [7, 8, 10, 19, 39–42]. This study includes a larger number of variables (47) including demographics, peri- and post-operative outcomes, surgical approaches, complications and mortality. These variables are used and compared in predicting LOS, mortality and readmission. Sets of key variables were identified using various data analysis techniques. Algorithms like Extra Tree Regressor, LASOO and correlation matrix with heat map help to extract essential features from all variables. Comparison between using all variables and selected variables has shown that the machine learning model performs better with all variables than the selected variables in predicting LOS, readmission and mortality prediction. This observation confirms the benefit of applying machine learning algorithms when high-dimensional data are available. In principle, a ML algorithm ought to be able to make use of all available information, giving a lower weighting to the less useful information.

Medical researchers have found ML algorithms helpful to predict the diagnosis and prognosis of various diseases and health conditions accurately [20, 21, 43, 44]. Moreover, a prediction model with a machine learning algorithm was used to detect early colorectal cancer and recurrence of stage IV colorectal cancer after tumour resection [23, 45]. However, prior studies related to patient LOS, readmission and mortality after colorectal surgery have been mainly limited to observation study of predictor variables, with little investigation of machine learning. The current study explores not only the significant predictor variables, but also investigates machine learning algorithms that can exploit them.

### LOS prediction

Pucciarelli et al., on their observational analysis, found median LOS is 13 days [7] in comparison to 11.26 days in this study. Kelly et al., in their research, found median LOS is 14 days for elective and 21 for emergency admissions [19]. Aravani et al. found that age, comorbidity, socioeconomic deprivation, stage of the disease, and emergency operations are better predictor variables of longer LOS [8]. Age, comorbidities, marital status and emergency readmission were linked to the likelihood of longer LOS [19]. Ahmed et al. showed that ASA grade, epidurals and oral opiates are associated with an earlier discharge [41]. Chiu et al. state that minor and major complications were better predictors of LOS than preoperative demographic and disease parameters [46]. Sex, congestive heart failure, weight loss, Crohn’s disease, preoperative albumin < 3.5 g/dL and hematocrit < 47%, baseline sepsis, ASA class ≥ 3, open surgery, surgical time ≥ 190 min, post-operative pneumonia, failure to wean from mechanical ventilation, deep venous thrombosis, urinary tract infection, systemic sepsis, surgical site infection and reoperation within 30-days from the primary surgery were the risk factors for prolonged LOS [47]. In contrast, this study found age, ASA grade, operative time, presence or absence of a stoma, robotic or laparoscopic approach to surgery, and complications are the significant predictor variables of LOS.

This study investigated the predictor variables’ scope in predicting LOS by building a prediction model with machine learning techniques, unlike the prior work. For LOS prediction, models were built using different machine learning algorithms, and the results were compared to find the best model. The model with SVR algorithms turns out to be the best and tuned further with a tuning algorithm. Finally, the adjusted model is used for LOS prediction, and the model predicted the LOS with an MAE and RMSE of 9.69 and 12.52, respectively. The LOS prediction regression outcome is then converted to a binary class with a few conditions mentioned in Sect. 3.1, which showed the model could predict LOS with 83.21% accuracy. Data analysis of different predictor variables of LOS shows that Age groups, ASA grade, Surgical approach, Operation mode, Complication and Robotic surgery are the most important predictor variables for LOS Prediction. On the other hand, the data analysis also shows that BMI is not a good predictor for LOS prediction.

### Readmission prediction

A national population-based study by Pucciarelli et al. found that gender, hospital location, comorbidities, type of surgery, stoma creation, open approach, rectal tumour location, and longer LOS were the predictor variables of 30-day readmission [7]. Chung et al. found that surgical site infection, hepatic disease, pulmonary disease, TNM stage, and operation time were the significant risk factors for readmission [48]. In contrast, this study found that Age, Surgical approach, Stoma formation, Preoperative nodal stage, Operation time, Operation mode, Previous surgery type, LOS, and the Specific procedure were the significant predictor variables for readmission.

A recent study by Rubens et al. created a risk model for predicting 30-day readmission rates after surgical treatment for colon cancer. Their model showed 60.2% accuracy, 58.8% sensitivity and 60.4% specificity with a limited number of variables [49]. In contrast, this study showed that the BI-LSTM algorithm predicts the readmission with an accuracy, sensitivity and specificity of 87.5%, 84.1% and 90.9%, respectively. Data analysis of the dataset found a 30-day readmission rate of 7.4%, which is comparable with the two recent reviews and meta-analyses findings ranged between 7 and 25% [50, 51].

### Post-operative mortality prediction

The risk of post-operative mortality after CRC has been investigated through several scoring systems [52–57]. To date, none of these scoring systems has been found effective as a predictor, and researchers have raised questions over their accuracy and usability [40, 58–60]. Moreover, these scoring systems require a high level of preoperative information, including laboratory values which may not always be available and thus they have not been employed widely [61]. Murray et al. found that age, ASA ≥ 3, renal failure, ascites, heart failure, disseminated cancer, hypoalbuminemia, open surgery, non-independent status and admission from a chronic care facility are the risk factors of 30-day mortality [40]. Wilkins et al. found that age, ASA grade IV–V, Dukes’ stage D, and urgent surgery are strongly associated with post-operative mortality. Their model predicted mortality with an area under the curve of 0.88 [39].

In contrast, this study considered post-operative mortality as a simple binary classification scenario. Prediction models were built and compared with machine-learning algorithms to predict mortality, 31 and 91 days mortality. Moreover, well-known evaluation metrics (i.e., accuracy, sensitivity and specificity) were used to evaluate the model performance. Machine learning algorithms were also compared in predicting mortality, 31 days mortality and 91 days mortality. The BI-LSTM algorithm model predicted the mortality and 91 days mortality with an accuracy of 80% and 94.2%, respectively and outperformed other algorithms. The random forest algorithm model outperformed other algorithms with an accuracy of 98.9% in predicting 31 days of mortality. The BI-LSTM model predicted the 31 days mortality with an accuracy of 96.6%.

Data analysis on mortality days shows that ASA grade, Complication, Curative Surgery, Additional procedures, Operation mode and Preoperative M stage are the variables that could be used to classify different groups of mortality days. BMI is not a good indicator in categorising different groups of mortality days. Figure  7 appears to show that a higher BMI leads to higher mortality days. However, this pattern is not correct as BMI groups 31–35 and ≤ 35 only represent around 5% of the whole dataset. Moreover, data analysis of the dataset shows a 30 days mortality of 3.39% for CRC surgery, which is comparable with the results of other similar studies ranged between 0.9 and 9.9% [7, 62, 63].

### Limitations

A limitation of this work has been the number of data samples, which were derived from a single centre. We are planning to collaborate with other colorectal groups to pool our data. Future work will therefore include a larger dataset comprising more samples and additional variables or features. Such additional variables may include nutritional status, obesity and metabolic syndrome, pre-operative heamoglobin and hypoalbuminemia, and comorbidities. Complications could be broken down into intra- and post-operative, along with a descriptor or Clavien-Dindo grade. It may also be informative to investigate the data patterns in colon and rectal cancers separately.

Nevertheless, some clear patterns have emerged from the data in the current study.

## Conclusions

Data analytics and AI have been shown to be accurate tools for predicting length of stay, readmission, and mortality following colorectal cancer surgery. Such predictive capability is important for designing the best patient care and prioritising resources.

These three proxies for patient outcomes were found to share the following common significant predictors: Age, Stoma, and Operation mode. LOS was also found to be a significant predictor for the other two patient outcomes, i.e., readmission and mortality. Other predictors had greater or lesser significance for each patient outcome. Nevertheless, the prediction algorithms were most effective when using the full data set rather than just the main predictors.

Bidirectional long short-term memory (BI-LSTM) was found to be the best prediction algorithm overall. In each case, we have demonstrated accuracies of greater than 80% and sensitivities and specificities of at least 84% and 75% respectively. The best results were achieved for 31 days mortality, with 96% accuracy, 93% sensitivity and 100% specificity. With improving techniques, richer data sets, and overlaid clinical expertise, further improvements can be anticipated, leading to improved patient outcomes and more efficient healthcare services.

## Data Availability

Enquiries about data access can be made via JK.

## Code availability

The computer code has been deposited in the github repository [25].
